# HLA-DR and HLA-DQ alleles in patients from the south of Brazil: markers for leprosy susceptibility and resistance

**DOI:** 10.1186/1471-2334-9-134

**Published:** 2009-08-22

**Authors:** Samira A da Silva, Priscila S Mazini, Pâmela G Reis, Ana M Sell, Luiza T Tsuneto, Paulo R Peixoto, Jeane EL Visentainer

**Affiliations:** 1 Departamento de Análises Clínicas, Universidade Estadual de Maringá, Paraná, Brazil; 2 15a Regional de Saúde da Secretaria de Saúde do Estado do Paraná, Maringá, Paraná, Brazil

## Abstract

**Background:**

Many epidemiological studies have shown that the genetic factors of the host play a role in the variability of clinical response to infection caused by *M. leprae*. With the purpose of identifying genes of susceptibility, the present study investigated the possible role of HLA-DRB1 and DQA1/DQB1 alleles in susceptibility to leprosy, and whether they account for the heterogeneity in immune responses observed following infection in a Southern Brazilian population.

**Methods:**

One hundred and sixty-nine leprosy patients and 217 healthy controls were analyzed by polymerase chain reaction amplification and reverse hybridization with sequence-specific oligonucleotide probes and sequence-specific primers(One Lambda^®^, CA, USA).

**Results:**

There was a positive association of HLA-DRB1*16 (*1601 and *1602) with leprosy *per se *(7.3% *vs*. 3.2%, *P *= 0.01, OR = 2.52, CI = 1.26–5.01), in accord with previous serological studies, which showed DR2 as a marker of leprosy. Although, HLA-DQA1*05 frequency (29.8% *vs*. 20.9%, *P *= 0.0424, OR = 1.61, CI = 1.09–2.39) was higher in patients, and HLA-DQA1*02 (3.0% *vs*. 7.5%, *P *= 0.0392, OR = 0.39, CI = 0.16 – 0.95) and HLA-DQA1*04 (4.0% *vs*. 9.1%, *P *= 0.0314, OR = 0.42, CI = 0.19 – 0.93) frequencies lower, *P*-values were not significant after the Bonferroni's correction. Furthermore, HLA-DRB1*1601 (9.0% *vs*. 1.8%; *P *= 0.0016; OR = 5.81; CI = 2.05–16.46) was associated with susceptibility to borderline leprosy compared to control group, and while HLA-DRB1*08 (11.2% *vs*. 1.2%; *P *= 0.0037; OR = 12.00; CI = 1.51 – 95.12) was associated with susceptibility to lepromatous leprosy, when compared to tuberculoid leprosy, DRB1*04 was associated to protection.

**Conclusion:**

These data confirm the positive association of HLA-DR2 (DRB1*16) with leprosy *per se*, and the protector effect of DRB1*04 against lepromatous leprosy in Brazilian patients.

## Background

Leprosy is a chronic infectious disease caused by the intracellular pathogen *Mycobacterium leprae *[1], whose clinical manifestations are largely confined to the skin and peripheral nervous system [2].

Although the global prevalence of leprosy has dropped dramatically, coinciding with the introduction of multiple-drug therapy, new-case detection rates have remained stable over the years at approximately 500,000 new cases per year. The majority of the remaining leprosy cases are found in India and a few other countries, including Brazil [3].

Considered a multifactorial disease, the expression of clinical manifestations of leprosy reflects the relationship between the host and the parasite. There is a strong correlation between cell-mediated immunity (CMI) against *M. leprae *antigens, observed *in vitro *and *in vivo*, and the clinical course of the disease. There are five clinical forms of the disease, ranging between two poles: tuberculoid (TT) and lepromatous (LL) leprosy. Tuberculoid leprosy is the result of high cell-mediated immunity, with a largely Th1-type immune response, and lepromatous leprosy is characterized by low cell-mediated immunity with a humoral Th2 response [2,4].

Many epidemiological studies, which had the objective of identifying genes of susceptibility [5], have shown that the genetic factors of the host play a role in the variability of clinical response to infection caused by both *M. tuberculosis *and *M. leprae*. The Major Histocompatibility Complex (MHC), located on chromosome 6p21, is the most polymorphic genetic system in mammalians, and has been studied with regard to a wide variety of diseases of distinct etiology, including infectious diseases. The fundamental role of the different molecules within the MHC is antigen processing and presentation to the T-cell receptor (TCR), which is crucial for the cell interactions in CMI [6].

In humans, while the classic class I *loci*, HLA-A, -B, and -C, bind peptides of intra-cellular origin and present them to CD8+T cells, resulting in the death of cells infected by *M. leprae*, the classic class II *loci*, HLA-DR, -DQ, and -DP, primarily bind peptides of extra-cellular origin and present them to CD4+T cells, resulting in cytokine production and T-cell help in antibody production.

While the idea of HLA-related genes being involved in the control of the clinical progression of leprosy is well documented, the possible role of the HLA region in susceptibility to infection *per se *has also been suggested [7]. Some results have already been found for class I HLA alleles [8], however a greater number of studies have reported increased frequencies of class II alleles in TT and LL patients in several populations [9-14].

The aims of this study were to determine which HLA class II polymorphisms affect differential susceptibility to leprosy and whether these account for the heterogeneity in immune responses observed following infection in a Southern Brazilian population.

## Methods

### Patients and controls

One hundred and sixty-nine (169) leprosy patients attended in the 15^a ^Regional de Saúde da Secretaria de Saúde, Paraná State, Southern Brazil, took part in this study. All the patients were residents in the cities of Maringá, Sarandi and Umuarama, and the surrounding region, in Paraná state. They were classified into four distinct groups according to clinical and laboratorial (Mitsuda's test and histology exams) observations made by the dermatologist responsible for leprosy diagnosis: TT (41 out of the 169 patients, 24.3%), LL (63 patients, 37.3%), Borderline (49 patients, 29.0%), and Indeterminate (4 patients, 2.4%).

Patient ages at the time of sample collection varied between 22 and 93 years (mean, 52.8; ± SD, 14.6). However, leprosy is traditionally a young person's disease, with a median age at onset of 35 years [15]. Seventy-two (42.6%) of the patients were female and 97 (57.4%) male.

The control group was composed of 217 healthy individuals, selected according to age, sex, ethnic group, occupation and other demographic parameters, with no history of HLA-associated diseases (hepatitis, auto-immune diseases, HIV and tuberculosis). They were typified by the Laboratório de Imunogenética of the Universidade Estadual de Maringá. Their ages varied from 20 to 58 years (mean, 42.0; SD, 7.1), and 106 (48.8%) were female and 111 (51.2%) male.

The population of Paraná is predominantly of European origin (80.6%), with a small but significant contribution of African (12.5%) and Amerindian (7.0%) genes [16]. Both the patients and healthy controls were classified as mixed ethnic groups, according to phenotypic characteristics, because according to Parra *et al*. (2003) [17], in Brazil, at an individual level, skin color determined by physical evaluation is a poor predictor of genomic African ancestry.

The risk of population stratification bias, due to differences in ethnic background between patients and controls, and variations of allele frequencies, according to ethnic background, were minimized by matching patients with control individuals of the same ethnic background, mean age, gender rates and residence in the same geographical areas of leprosy prevalence.

All the participants signed written informed-consent forms, authorizing the use of their samples in the study, which was approved by the Ethics Committee for Human Research at the Universidade Estadual de Maringá.

### Determination of *HLA *class II alleles

Blood samples (10 mL) were collected from the subjects in tubes containing anticoagulant (EDTA) and centrifuged at 2,500 rpm for 15 minutes, and the Buffy-coat was conserved at -20°C until use. The genomic DNA was extracted by EZ-DNA kits (Biological Industries^®^, Kibbutz Beit Haemek, Israel) from 150 μL of frozen blood, and then stored at -20°C.

HLA class II alleles for DRB1* (alleles 01–16), DQA1* (alleles 01–06) and DQB1* (alleles 02–06) were genotyped by combining PCR-SSP (polymerase chain reaction – sequence-specific primers), using the HLA genotyping kit [18] with modifications [19] and/or PCR-SSO (polymerase chain reaction – sequence-specific oligonucleotides) using Luminex (One Lambda^®^, CA, USA). High resolution PCR-SSP typing (One Lambda^®^, CA, USA) was also used to define the DRB1*04 (0401–0463) and HLA-DRB1*16 (alleles 1601–1608) alleles, as these *loci *are associated with leprosy.

### Statistical analysis

Allele frequency was obtained through direct counting of the alleles. The allele-frequency distributions were confirmed as being in Hardy-Weinberg equilibrium by calculating the expected frequencies of the genotypes and comparing them to the observed values using Arlequin software, version 3.1 [20], available at http://cmpg.unibe.ch/software/arlequin3.

The analysis of the associations between the HLA variables and the occurrence of leprosy *per se *and the LL and TT subgroups, was performed using the Chi square test, with the Yates's correction or Fisher's Exact Test when necessary, using the Graph Pad Software program (San Diego, CA, USA), available at http://www.graphpad.com/quickcalcs/contingency1.cfm. Data were considered statistically significant when the *P *value was less than or equal to 0.05. However, to account for multiple comparisons, the observed *P *values were corrected (pc) for the number of alleles when one locus was considered alone.

The odds ratios (OR), with 95% confidence intervals (95% CI), were calculated using SISA statistics online http://home.clara.net/sisa/, to evaluate the risk of the individual developing the disease while having a particular HLA type.

## Results

The possibility of an association between class II HLA alleles and leprosy in a population from the south of Brazil was investigated in this study through the comparison of the HLA-DRB1, DQA1 and DQB1 allele frequencies of patients suffering the disease with those of a healthy control group.

### Leprosy *per se vs*. control group

The HLA-DRB1 and DQA1\DQB1 allele frequencies of the patients and control group are shown in Tables 1 and 2, respectively. There were differences between leprosy *per se *patients and the control group for HLA-DRB1*16, which appeared with greater frequency in the patients (7.3% *vs*. 3.2%, *P *= 0.01, OR = 2.52, CI = 1.26–5.01, pc = 0.13). Among the DRB1*16 alleles, DRB1*1602 had the greatest frequency (3.3% *vs*. 1.1%, *P *= 0.0684). HLA-DQA1*05 frequency (29.8% *vs*. 20.9%, *P *= 0.0424, OR = 1.61, CI = 1.09–2.39, pc = 0.4664) was higher in patients compared to the control group, while HLA-DQA1*02 (3.0% *vs*. 7.5%, *P *= 0.0392, OR = 0.39, CI = 0.16–0.95, pc = 0.4312), and DQA1*04 (4.0 *vs*. 9.1, *P *= 0.0314, OR = 0.42, CI = 0.19–0.93, pc = 0.3454) frequencies were lower. HLA-DQB1*03 showed a tendency to protection against leprosy *per se *(27.3% *vs*. 33.7%, *P *= 0.0684).

**Table 1:** Frequencies of HLA-DRB1 alleles in leprosy *per se *patients and control group

Alleles	Leprosy *per se*		Controls		*P*	OR	CI 95%
	(N = 169)		(N = 217)				
				
	n	%	n	%			
DRB1*01	30	8.8	38	8.8	1		
DRB1*03	35	10.3	52	12.0	0.4641		
DRB1*04	23	6.8	42	9.7	0.1701		
DRB1*07	35	10.3	50	11.5	0.6217		
DRB1*08	25	7.3	24	5.5	0.2847		
DRB1*09	11	3.2	7	1.6	0.1487		
DRB1*10	9	2.66	7	1.6	0.3169		
DRB1*11	35	10.3	59	13.6	0.1528		
DRB1*12	4	1.1	5	1.2	1		
DRB1*13	38	11.2	69	15.9	0.0512		
DRB1*14	13	3.8	19	4.4	0.8527		
DRB1*15	50	14.7	48	11.1	0.1002		
DRB1*16	25	7.3	14	3.2	0.01*	2.52	1.26–5.01
DRB1*1601	13	3.8	8	1.8	0.1126		
DRB1*1602	11	3.3	5	1.1	0.0684		
DRB1*1605	1	0.2	0	0	0.4378		
DRB1*1608	0	0	1	0.2	1		

N: number of individuals. n: number of alleles. *P *: probability. OR: odds ratio. CI: confidence interval. *pc: 0.13.

**Table 2:** Frequencies of HLA-DQA1 e DQB1 alleles in leprosy *per se *patients and control group

Alleles	Leprosy *per se*		Controls		*P*	OR	CI 95%
	(N = 98)		(N = 178)				
				
	n	%	n	%			
DQA1*01	94	47.5	171	45.7	0.9866		
DQA1*02	6	3.0	28	7.5	0.0392*	0.39	0.16–0.95
DQA1*03	17	8.6	43	11.5	0.2770		
DQA1*04	8	4.0	34	9.1	0.0314**	0.42	0.19–0.93
DQA1*05	59	29.8	78	20.9	0.0424***	1.61	1.09–2.39
DQA1*06	2	1.0	7	1.9	0.5024		
	N = 102		N = 184				

DQB1*02	39	19.7	59	15.8	0.4110		
DQB1*03	54	27.3	126	33.7	0.0684		
DQB1*04	11	5.6	19	5.1	0.9063		
DQB1*05	40	20.2	66	17.6	0.7032		
DQB1*06	53	26.8	104	27.8	0.6258		

N: number of individuals. n: number of alleles. *P*: probability. OR: odds ratio. CI: confidence interval. *pc: 0.4312. **pc: 0.3454. ***pc: 0.4664.

### Leprosy subgroups *vs*. control group

Table 3 shows the significant differences in HLA frequencies between the control group and the LL, Borderline, and TT leprosy subtype patients. There were differences in allelic frequencies between LL patients and the control group: while DRB1*16 (7.9% *vs*. 3.2%, *P *= 0.0405, OR = 2.59, CI = 1.12–5.97) was associated with susceptibility to the LL form, DRB1*04 (3.2% *vs*. 9.7%, *P *= 0.0165, OR = 0.31, CI = 0.11–0.87) was associated with protection against it. On further, high-resolution, analysis of the DRB1*04 and DRB1*16 alleles, only DRB1*1602 frequency was higher in LL patients than in the control group (4.8% *vs*. 1.2%, *P *= 0.0197, OR = 4.29, CI = 1.29–14.30). HLA-DQA1*03 frequency was lower (3.8% *vs*. 11.5%, *P *= 0.0184, OR = 0.25, CI = 0.07–0.87), while DQA1*05 (32.5% *vs*. 20.9%, *P *= 0.0220, OR = 2.38, CI = 1.17–4.86) and DQB1*02 (27.5% *vs*. 15.8%, *P *= 0.0103, OR = 2.59, CI = 1.29–5.1) frequencies were higher in LL patients compared to the control group.

**Table 3:** Frequencies of HLA class II alleles in leprosy subgroups and control group

Alleles	Lepromatous		Controls				
	(N = 63)		(N = 217)		*P*	OR	CI 95%
	
	n	%	n	%			
DRB1*04	4	3.2	42	9.7	0.0165	0.31	0.11–0.87
DRB1*16	10	7.9	14	3.2	0.0405	2.59	1.12–5.97
DRB1*1602	6	4.8	5	1.2	0.0197	4.29	1.29–14.30
	N = 40		N = 178				
DQA1*03	3	3.8	43	11.5	0.0184	0.25	0.07–0.87
DQA1*05	26	32.5	78	20.9	0.0220	2.38	1.17–4.86
	N = 40		N = 184				
				
DQB1*02	22	27.5	59	15.8	0.0103	2.59	1.29–5.19
	Borderline		Controls				
	(N = 44)		(N = 217)				
				
DRB1*09	6	6.8	7	1.6	0.0112	4.74	1.51–14.87
DRB1*16	8	9.0	14	3.2	0.0175	3.22	1.26–8.23
DRB1*1601	8	9.0	8	1.8	0.0016*	5.81	2.05–16.46
	N = 30		N = 178				
				
DQA1*04	1	1.7	34	9.1	0.0337	0.15	0.02–1.11
	Tuberculoid		Controls				
	(N = 45)		(N = 217)				
				
DRB1*1602	4	4.4	5	1.1	0.0498	4.14	1.07–16.06
	N = 24		N = 178				
				
DQA1*02	0	0	28	7.5	0.0298	0.23	0.03–1.8
DQA1*05	18	37.5	78	20.9	0.0046	3.85	1.46–10.15

N: number of individuals. n: number of alleles. *P*: probability. OR: odds ratio. CI: confidence interval. *pc: 0.0208.

HLA-DRB1*09 (6.8 *vs*. 1.6%, *P *= 0.0112, OR = 4.74, CI = 1.51–14.87) and DRB1*16 (9.0% *vs*. 3.2%, *P *= 0.0175, OR = 3.22, CI = 1.26–8.23) frequencies were higher in Borderline patients than in the control group. On further, high-resolution, analysis, only DRB1*1601 frequency was higher in these patients (9.0% *vs*. 1.8%, *P *= 0.0016, OR = 5.81, CI = 2.05–16.46, pc = 0.0208). HLA-DQA1*04 frequency was lower in Borderline patients than in the control group (1.7% *vs*. 9.1%, *P *= 0.0337, OR = 0.15, CI = 0.02–1.11).

In patients with TT leprosy, although DRB1*16 frequency was not different to that of the control group, the DRB1*1602 allele frequency was found to be higher (4.4% *vs*. 1.1%, *P *= 0.0498, OR = 4.14, CI = 1.07–16.06). Furthermore, HLA- DQA1*05 frequency was higher (37.5% *vs*. 20.9%, *P *= 0.0046, OR = 3.85, CI = 1.46–10.15), while DQA1*02 frequency was lower (0% *vs*. 7.5%, *P *= 0.0298, OR = 0.23, CI = 0.03–1.80) in these patients.

### Lepromatous leprosy *vs*. Tuberculoid leprosy

When comparing the opposite poles of the disease (LL *vs*. TT) (Table 4), a difference was observed for HLA-DRB1*04 (3.2% *vs*. 12.8%; *P *= 0.0090; OR = 0.20; CI = 0.06–0.67, pc = 0.117), which was associated with protection against the LL form. HLA-DRB1*08 was associated with a predisposition to LL leprosy (11.2% *vs*. 1.2%; *P *= 0.0037; OR = 12.00; CI = 1.51 – 95.12, pc = 0.0481), and HLA-DRB1*14 showed a tendency towards association with these patients (1.5% *vs*. 6.9%; *P *= 0.0595).

**Table 4:** HLA-DRB1 allele frequencies in Lepromatous (LL) and Tuberculoid (TT) leprosy patients

	LL		TT				
Alleles	(N = 63)		(N = 43)		*P*	OR	CI 95%
				
	n	%	n	%			
DRB1*01	12	9.5	11	12.8	0.5745		
DRB1*03	13	10.3	13	15.1	0.3693		
DRB1*04	4	3.2	11	12.8	0.0090*	0.20	0.06–0.67
DRB1*07	14	11.2	9	10.4	0.8741		
DRB1*08	14	11.2	1	1.2	0.0037**	12.00	1.51–95.12
DRB1*09	4	3.2	2	2.3	1.0000		
DRB1*10	4	3.2	1	1.2	0.6462		
DRB1*11	11	8.7	11	12.8	0.4422		
DRB1*12	2	1.5	1	1.2	1.0000		
DRB1*13	14	11.2	7	8.1	0.6131		
DRB1*14	2	1.5	6	6.9	0.0595		
DRB1*15	21	16.6	9	10.5	0.2410		
DRB1*16	10	7.9	5	5.8	0.5850		
DRB1*1601	3	2.4	0	0	0.2698		
DRB1*1602	6	4.8	5	5.8	0.7544		

N: number of individuals. n: number of alleles. *P*: probability. OR: odds ratio. CI: confidence interval. * pc: 0.117. **pc: 0.0481.

No statistically significant difference in the allele frequency distributions was observed between LL and TT forms for HLA-DQA and DQB alleles (data not shown).

## Discussion

This case-control study investigated the genetic variation present in HLA-DRB1, DQA1 and DQB1 genes, and its relation to leprosy in Southern Brazil.

In infection caused by *M. leprae*, HLA alleles influence not only susceptibility or resistance to the disease, but also the course of the disease. The main role of HLA molecules is to present peptides derived from *M. leprae *to the T cells of the host. Susceptibility to an infectious disease may be due to imperfections in some stages of this system. An individual that has a particular combination of HLA alleles that are not linked to the peptide in an appropriate way, or for whom the HLA-peptide linkage does not develop a proper lymphocyte response, will be less able to resist the invasion of the infectious agent than an individual that does not have such deficiencies [21]. In patients whose HLA systems offer protection against the disease, these genes probably select and stimulate T cells that multiply and eliminate the agent with inflammatory cytokine production, or else destroy the infected cells [22].

Several studies identify a consistent form for the participation of HLA alleles and haplotypes, especially class II genes, as an important genetic factor associated to leprosy. The clinical manifestation of the disease depends on the type of immune response shown by the host, and exchanges between Th1 and Th2 response types may be partially controlled by a mechanism of antigen presentation involving HLA molecules [23]. In the TT form of the disease, the production of proinflammatory cytokines by Th1 cells may help in the clearance of the bacillus. However, in the LL form, the immunosuppressor cytokines produced by the Th2 response may make this response difficult.

In the present study, HLA-DRB1*16, a subtype of DR2, participates as a susceptibility marker for leprosy *per se*, confirming previous serological results that showed an association between HLA-DR2 and leprosy *per se *in patients from an equivalent geographical area (South Brazil) [11]. The association of HLA-DR2 (now DRB1*15 and DRB1*16) with susceptibility to leprosy *per se *has been highlighted in other studies [24,25] and in LL and TT patients, compared to healthy controls, in many populations around the world (Surinam, India, Venezuela, Egypt, Chine, Japan, Korea, Mexico, Turkey, Brazil) [reviewed in [12,26]]. Associations with the class II region are thought to occur due to the class II restriction of the presentation of mycobacterial epitopes to T-helper cells.

Rani et al. (1992), in Korean, showed positive associations of HLA-DR2 and DQ1 with LL leprosy, and DR9 and DQ7 with Borderline leprosy; and negative associations between DR4 and DQ3 and LL leprosy [7]. The present study is in accord with these results: HLA-DRB1*DR9 was associated with susceptibility to Borderline leprosy and HLA-DRB1*04 was associated with protection against LL leprosy, as a lower frequency was observed in these patients compared to in the TT group, and DQB1*03 showed a tendency towards protection. Strong linkage disequilibria, in some populations, are known to exist within the MHC (gene order: HLA-DQB1, DQA1, DRB1) [27-29]. The weak positive association of DQB1*03 with protection against leprosy (Table 2) may be due to the strong linkage disequilibrium between DRB1*04 and this DQ allele.

Similar results for DRB1*04 were reported by Joko *et al*. (2000) for leprosy *per se *in a Japanese population [30] and, more recently, by both Vanderborght *et al*. (2007) in both Brazilian and Vietnamese populations [14] and Motta *et al*. (2007) in an Argentinean population [31].

In addition, according to the data of the present study, HLA-DRB1*08 frequency was higher in lepromatous patients than tuberculoid patients, indicating a role in susceptibility to the most severe form of leprosy.

Rani *et al*. (1993) found a significantly higher frequency of DQB1*0601 in leprosy patients than in healthy controls, while DQA1*0103 was most frequent in LL patients and DQA1*0102 was selectively higher in borderline lepromatous patients [32]. On the other hand, DRB1*0701, DQB1*0201 and DQA1*0201 frequencies were all lower in multibacillary leprosy patients compared to TT patients and controls, and DQB1*0503 was selectively lower in TT patients. In the present study, HLA-DQA1*05 frequency was significantly higher in leprosy patients, while DQA1*02 and *04 frequencies were lower.

An unrelated genome-wide scan in 71 multi-case families from Brazil found chromosome region 6p21 (lod = 3.23) to be weakly linked to leprosy [33]. More detailed analysis indicated that more than one *locus *in each of these dense immune-response gene regions might affect susceptibility to leprosy, including genes that contribute to leprosy *per se *and to lepromatous, as opposed to tuberculoid, disease subtypes [13]. Early candidate-gene analysis, usually involving the HLA region on chromosome 6p21, has provided the first experimental evidence for the complex nature of the genetic variations involved in host genetic susceptibility to leprosy. It is therefore necessary that research be continued via a study with a larger number of patients in each group in order to verify the genes that are possibly related to protection against and susceptibility to leprosy, and to disease progression, in this Brazilian population.

## Conclusion

Although there was great genotypic variety in the Brazilian population, the results of the present study confirm the significant participation of HLA-DR2 (DRB1*16) and DQA1 in susceptibility to leprosy *per se*, and the participation of HLA-DRB1*04 in protection and of DRB1*08 in susceptibility to the severe form of the disease (lepromatous leprosy). Therefore, HLA-DRB1*1601 was associated with susceptibility to Borderline leprosy. Taken together, these HLA analyses suggest the possibility of there being more than one *locus *of leprosy susceptibility in the MHC.

## Competing interests

The authors declare that they have no competing interests.

## Authors' contributions

PSM, SAS and PGR carried out the molecular genetic studies. PSM participated in the design of the study. JELV, PSM and AMS performed the statistical analysis. JELV conceived of the study, and participated in its design and coordination and helped to draft the manuscript. PRP participated in the clinical diagnostic of leprosy. All authors read and approved the final manuscript.

## Pre-publication history

The pre-publication history for this paper can be accessed here:

http://www.biomedcentral.com/1471-2334/9/134/prepub
